# Identification of Robust Pathway Markers for Cancer through Rank-Based Pathway Activity Inference

**DOI:** 10.1155/2013/618461

**Published:** 2013-02-27

**Authors:** Navadon Khunlertgit, Byung-Jun Yoon

**Affiliations:** Department of Electrical and Computer Engineering, Texas A&M University, College Station, TX 77843-3128, USA

## Abstract

One important problem in translational genomics is the identification of reliable and reproducible markers that can be used to discriminate between different classes of a complex disease, such as cancer. The typical small sample setting makes the prediction of such markers very challenging, and various approaches have been proposed to address this problem. For example, it has been shown that pathway markers, which aggregate the gene activities in the same pathway, tend to be more robust than gene markers. Furthermore, the use of gene expression ranking has been demonstrated to be robust to batch effects and that it can lead to more interpretable results. In this paper, we propose an enhanced pathway activity inference method that uses gene ranking to predict the pathway activity in a probabilistic manner. The main focus of this work is on identifying robust pathway markers that can ultimately lead to robust classifiers with reproducible performance across datasets. Simulation results based on multiple breast cancer datasets show that the proposed inference method identifies better pathway markers that can predict breast cancer metastasis with higher accuracy. Moreover, the identified pathway markers can lead to better classifiers with more consistent classification performance across independent datasets.

## 1. Introduction

Advances in microarray and sequencing technologies have enabled the measurement of genome-wide expression profiles, which have spawned a large number of studies aiming to make accurate diagnosis and prognosis based on gene expression profiles [1–4]. For example, there has been significant amount of work on identifying markers and building classifiers that can be used to predict breast cancer metastasis [2, 4]. Many existing methods have directly employed gene expression data without any knowledge of the interrelations between genes. As a result, the predicted gene markers often lack interpretability and many of them are not reproducible in other independent datasets.

To overcome this problem, several different approaches have been proposed so far. For example, a recent work by Geman et al. [3] proposed an approach that utilizes the relative expression between genes, rather than their absolute expression values. It was shown that the resulting markers are easier to interpret, robust to chip-to-chip variations, and more reproducible across datasets. Another possible way to address the aforementioned problem is to interpret the gene expression data at a “modular” level through data integration [5–11]. These methods utilize additional data sources and prior knowledge—such as protein-protein interaction (PPI) data and pathway knowledge—to jointly analyze the expression of interrelated genes. This results in modular markers, such as *pathway markers* and *subnetwork markers*, which have been shown to improve the classification performance and also to be more reproducible across independent datasets [8–11]. In order to utilize pathway markers, we need to infer the pathway activity by integrating the gene expression data with pathway knowledge. For example, Guo et al. [6] used the mean or median expression value of the member genes (that belong to the same pathway) as the activity level of a given pathway. Recently, Su et al. [10] proposed a probabilistic pathway activity inference method that uses the log-likelihood ratio between different phenotypes based on the expression level of each member gene.

In this work, we propose an enhanced pathway activity inference method that utilizes the ranking of the member genes to predict the pathway activity in a probabilistic manner. The immediate goal is to identify better pathway markers that are more reliable, more reproducible, and easier to interpret. Ultimately, we aim to utilize these markers to build accurate and robust disease classifiers. The proposed method is motivated by the relative gene expression analysis strategy proposed in [3, 12] and it builds on the concept of probabilistic pathway activity inference proposed in [10, 11]. In this study, we focus on predicting breast cancer metastasis and demonstrate that the proposed method outperforms existing methods. Preliminary results of this work have been originally presented in [13].

## 2. Materials and Methods

### 2.1. Study Datasets

Six independent breast cancer microarray gene expression datasets have been used in this study: GSE2034 (USA) [4], NKI295 (The Netherlands) [14], GSE7390 (Belgium) [15], GSE1456 (Stockholm) [16], GSE15852, and GSE9574. The Netherlands dataset uses a custom Agilent chip and it has been obtained from the Stanford website [17]. All datasets have been profiled using the Affymetrix U133a platform and they have been downloaded from the Gene Expression Omnibus (GEO) website [18].

The above datasets have been used in our study both with and without (re)normalization. To test the reproducibility of pathway markers, we selected the USA dataset and the Belgium dataset, both of which were obtained using the Affymetrix platform. The raw data for these two datasets have been normalized by utilizing the microarray preprocessing methods provided in the Bioconductor package [19]. We applied three popular normalization methods—RMA, GCRMA, and MAS5—with default setting.

The pathway data have been obtained from the MSigDB 3.0 Canonical Pathways [20]. This pathway dataset consists of 880 pathways, where 3,698 genes in these pathways intersect with all datasets.

### 2.2. Gene Ranking

In this study, we utilize “gene ranking” or the relative ordering of the genes based on their expression levels within each profile [3]. Consider a pathway that contains *n* member genes *G* = {*g*_1_, *g*_2_,…, *g*_*n*_} after removing the genes that are not included in all datasets. Given a sample **x**_*k*_ = {*x*_*k*_^1^, *x*_*k*_^2^,…, *x*_*k*_^*n*^} that contains the expression level of the member genes, the gene ranking **r**_*k*_ is defined as follows:

(1) $$\begin{array}{c} r_{k}=\left\{r_{k}^{i,j}\;|\;1\leq i<j\leq n\right\}, \end{array}$$

where

(2) $$\begin{array}{c} r_{k}^{i,j}=\{\begin{array}{cc} 1, & \text{if}\;\;x_{k}^{i}<x_{k}^{j},\\ 0, & \text{otherwise}. \end{array} \end{array}$$

The resulting gene ranking **r**_*k*_ is a binary vector representing the ordering of the member genes based on their expression values in the *k*th sample **x**_*k*_. To preserve the gene ranking in each sample, we do not employ any between-sample normalization.

### 2.3. Pathway Activity Inference Based on Gene Ranking

To infer the pathway activity, we follow the strategy proposed in [10], where the activity level *a*_*k*_ of a given pathway in the *k*th sample is predicted by aggregating the probabilistic evidence of all the member genes. The main difference between the strategy proposed in this work and the original strategy [10] is that we estimate the probabilistic evidence provided by each gene based on its ranking rather than its expression value. More specifically, the pathway activity level is given by

(3) $$\begin{array}{c} a_{k}=\sum_{1\leq i<j\leq n}\lambda_{i,j}\left(r_{k}^{i,j}\right), \end{array}$$

where *λ*_*i*,*j*_(*r*_*k*_^*i*,*j*^) is the log-likelihood ratio (LLR) between the two phenotypes (i.e., class labels) for the ranking **r**_*k*_. The LLR *λ*_*i*,*j*_(*r*_*k*_^*i*,*j*^) is defined as

(4) $$\begin{array}{c} \lambda_{i,j}\left(r_{k}^{i,j}\right)=\log\left[\frac{f_{i,j}^{1}\left(r_{k}^{i,j}\right)}{f_{i,j}^{2}\left(r_{k}^{i,j}\right)}\right], \end{array}$$

where *f*_*i*,*j*_^1^(*r*) is the conditional probability mass function (PMF) of the ranking of the expression level of gene *g*_*i*_ and gene *g*_*j*_ under phenotype 1 and *f*_*i*,*j*_^2^(*r*) is the conditional PMF of the ranking of the expression level of gene *g*_*i*_ and gene *g*_*j*_ under phenotype 2.

In practice, the number of possible gene pairs $\left(\begin{array}{c} n\\ 2 \end{array}\right)$ may be too large when we have large pathways with many member genes (i.e., when *n* is large). To reduce the computational complexity, we prescreen the gene pairs based on the mutual information [21] as follows. For every gene pair (*i*, *j*), we first compute the mutual information between the ranking *r*_*k*_^*i*,*j*^ and the corresponding phenotype *c*_*k*_. Then we select the top 10% gene pairs with the highest mutual information and use only these gene pairs for computing the pathway activity level defined in (3). Although we selected the top 10% gene pairs for simplicity, this may not be necessarily optimal and one may also think of other strategies for adaptively choosing this threshold.

**Figure 1:**
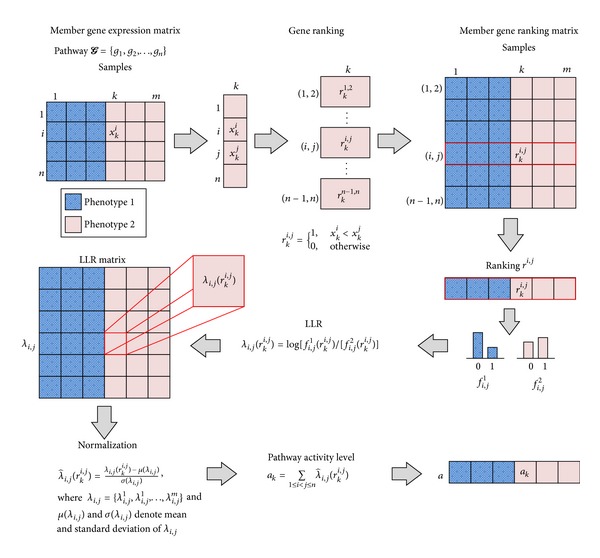
Probabilistic inference of rank-based pathway activity. For a given pathway, we first compute the ranking of the member genes for each individual sample in the dataset. Then we estimate the conditional probability mass function (PMF) of the gene ranking under each phenotype. Next, we transform the gene ranking into log-likelihood ratios (LLRs) based on the estimated PMFs and normalize the LLR matrix. Finally, the pathway activity level is inferred by aggregating the normalized LLRs of the member genes.

In a practical setting, we may not have enough training data to reliably estimate the PMFs *f*_*i*,*j*_^1^(*r*) and *f*_*i*,*j*_^2^(*r*). For this reason, we normalize the original LLR *λ*_*i*,*j*_(*r*_*k*_^*i*,*j*^) as follows to decrease its sensitivity to small alterations in gene ranking:

(5) $$\begin{array}{c} {\hat{\lambda }}_{i,j}\left(r_{k}^{i,j}\right)=\frac{\lambda_{i,j}\left(r_{k}^{i,j}\right)-\mu \left(\lambda_{i,j}\right)}{\sigma \left(\lambda_{i,j}\right)}, \end{array}$$

where *μ*(*λ*_*i*,*j*_) and *σ*(*λ*_*i*,*j*_) are the mean and standard deviation of *λ*_*i*,*j*_(*r*_*k*_^*i*,*j*^) across all *k* = 1,…, *n*.  Figure 1 illustrates the overall process.

### 2.4. Assessing the Discriminative Power of Pathway Markers

In order to assess the discriminative power of a pathway marker, we compute the *t*-test statistics score, which is given by

(6) $$\begin{array}{c} t\left(a\right)=\frac{\mu_{1}-\mu_{2}}{\sqrt{\sigma_{1}/K_{1}+\sigma_{2}/K_{2}}}, \end{array}$$

where **a** = {*a*_*k*_} is the set of inferred pathway activity levels for a given pathway, *μ*_*ℓ*_ and *σ*_*ℓ*_ represent the mean and the standard deviation of the pathway activity levels for samples with phenotype *ℓ* ∈ {1,2}, respectively, and *K*_*ℓ*_ represents the number of samples in the dataset with phenotype *ℓ*. This measure has been widely used in previous studies to evaluate the performance of pathway markers [9, 10].

### 2.5. Evaluation of the Classification Performance

In order to evaluate the classification performance, we use the AUC (Area under ROC Curve). Many previous studies [8–11] have utilized AUC due to its ability to summarize the efficacy of a classification method over the entire range of specificity and sensitivity. We compute the AUC based on the method proposed in [22]. Given a classifier, let *x*_1_, *x*_2_,…, *x*_*m*_ be the output of the classifier for *m* positive samples and let *y*_1_, *y*_2_,…, *y*_*n*_ be the output for *n* negative samples. The AUC of the classifier can be computed as follows:

(7) $$\begin{array}{c} A=\frac{1}{mn}\sum_{i=1}^{m}\sum_{j=1}^{n}I\left(x_{i}>y_{j}\right), \end{array}$$

where

(8) $$\begin{array}{c} I\left(x_{i}>y_{j}\right)=\{\begin{array}{cc} 1, & \text{if}\;\;x_{i}>y_{j},\\ 0, & \text{otherwise}. \end{array} \end{array}$$

## 3. Results and Discussion

### 3.1. Discriminative Power of the Pathway Markers Using the Proposed Method

In order to assess the performance of the rank-based pathway activity inference method proposed in this paper, we first evaluated the discriminative power of the pathway markers following a similar setup that was adopted in a number of previous studies [9, 10]. For comparison, we also evaluated the performance of the mean and median-based schemes proposed in [6] and the original probabilistic pathway activity inference method (we refer to this method as the “LLR method” for simplicity) presented in [10]. As explained in Materials and Methods, the discriminative power of a pathway marker was measured based on the absolute *t*-test score of the inferred pathway activity level. Then the pathway markers were sorted according to their *t*-score, in a descending order.

**Figure 2:**
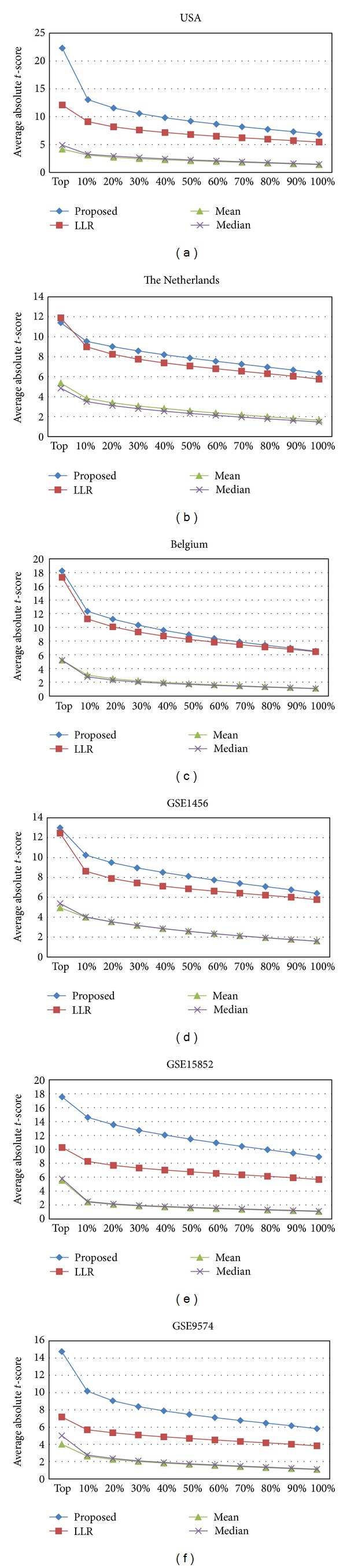
Discriminative power of pathway markers. We computed the mean absolute *t*-score of the top *P*% markers for each dataset without any further normalization.

Figure 2 shows the discriminative power of the pathway markers on the six datasets using different activity inference methods. On each dataset, we computed the mean absolute *t*-test statistics score of the top *P*% pathways for each of the four pathway activity inference methods. The *x*-axis corresponds to the proportion (*P*%) of the top pathway markers that were considered and the *y*-axis shows the mean absolute *t*-test score for these pathway markers. As we can see from Figure 2, the proposed method clearly improves the discriminative power of the pathway markers on all six datasets that we considered in this study. In order to investigate the effect of normalization on the discriminative power of the pathway activity inference methods, we repeated this experiment using the USA and the Belgium datasets, where we first normalized the raw data using three different normalization methods (RMA, GCRMA, and MAS5) and then evaluated the discriminative power of the pathway markers. The results are summarized in Figure S1 (see Supplementary Material available online at http://dx.doi.org/10.1155/2013/618461), where we can see that the proposed rank-based scheme is not very sensitive to the choice of the normalization method and performs consistently well in all cases.

**Figure 3:**
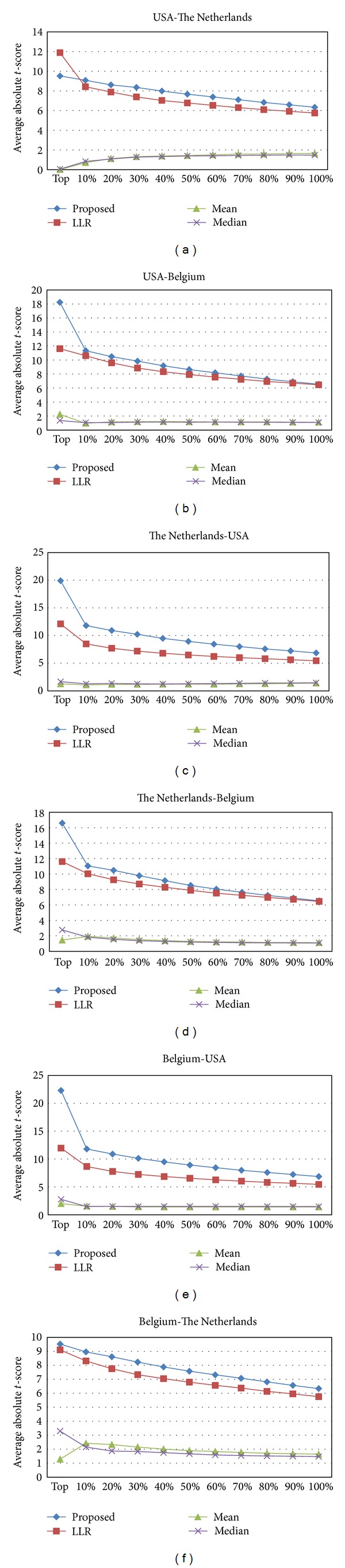
Discriminative power of pathway markers across different datasets. The pathway markers have been ranked and sorted using the first dataset, and their discriminative power has been reevaluated using the second dataset. As before, the mean absolute *t*-score was used for assessing the discriminative power.

Next, we investigated how the top pathway markers identified on a specific dataset perform in other independent datasets. We first ranked the pathway markers based on their mean absolute *t*-test statistics score in one of the datasets and then estimated the discriminative power of the top *P*% markers on a different dataset. These results are shown in Figure 3, where the first dataset is used for ranking the markers and the second dataset is used for assessing the discriminative power. As we can see from Figure 3, the pathway markers identified using the mean- and the median-based schemes do not retain their discriminative power very well in other datasets. Both the LLR method [10] and the proposed rank-based inference method perform well across different datasets, where the proposed method clearly outperforms the previous LLR method. It is interesting to see that the discriminative power of the markers is retained even when we consider datasets that are obtained using different platforms. For example, USA/Belgium datasets are profiled on the U133a platform and The Netherlands dataset is profiled on a custom Agilent chip, but Figure 3 shows that pathway markers identified using the proposed method retain their discriminative power across these datasets. As before, we repeated these experiments after normalizing the datasets using different normalization methods. The results are depicted in Figure S2, where we can see that the proposed method works very well, regardless of the normalization method that was used. Interestingly, this is also true even when the first dataset and the second dataset are normalized using different methods, as shown in Figures S3 and S4.

Another interesting observation is that the rank-based method can overcome one of the limitations of the previous LLR method. For example, normalization of the Belgium dataset using GCRMA results makes the LLR method fail, as some of the genes loose variability and some of the LLR values become infinite. We can see this issue in Figures S1(d), S2(c), S3(a), and S3(f). However, this limitation is easily overcome by the proposed method through the use of gene ranking and the preselection of informative gene pairs based on mutual information.

### 3.2. Classification Performance of the Pathway Markers Using the Proposed Method

**Figure 4:**
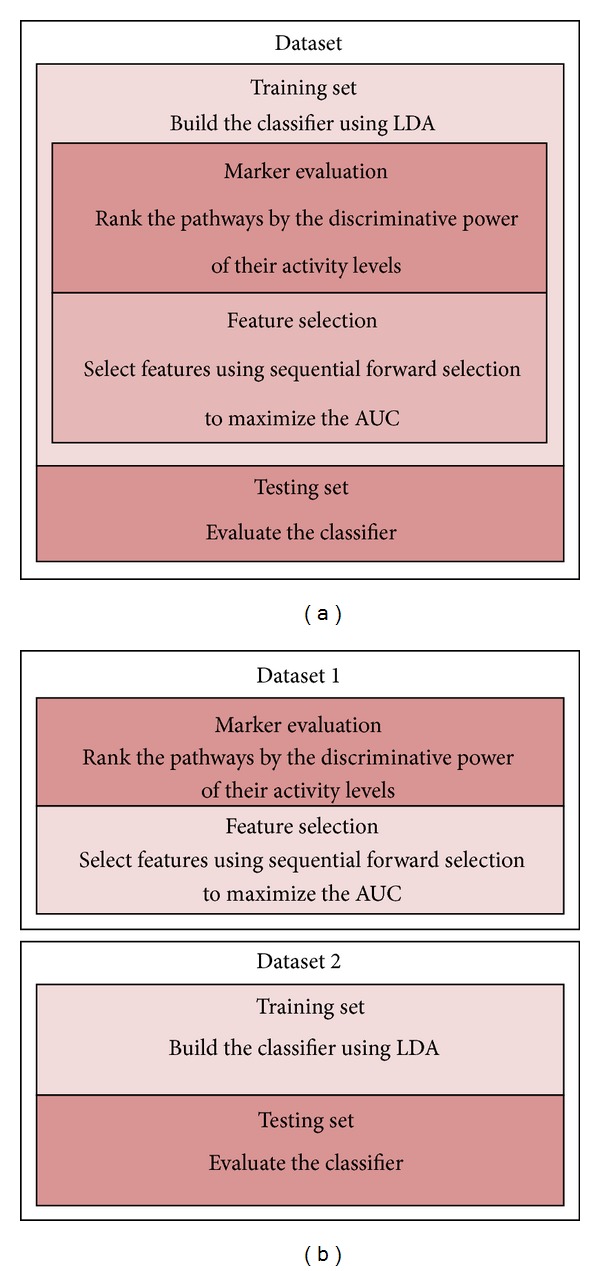
Experimental setup for evaluating the classification performance. (a) The setup for the within-dataset experiment. (b) The setup for the cross-dataset experiment.

Next, we evaluated the classification performance of the proposed rank-based pathway activity inference method. For this purpose, we performed fivefold cross validation experiments, following a similar setup used in previous studies [8–11]. We first performed the within-dataset experiments for each of the six datasets. First, a given dataset was randomly divided into fivefolds, where fourfolds (training dataset) were used for constructing an LDA (Linear Discriminant Analysis) classifier and the remaining fold (testing dataset) was used for evaluating its performance. To construct the classifier, the training dataset was again divided into threefolds, where twofolds (marker-evaluation dataset) were used for evaluating the pathway markers and the remaining onefold (feature-selection dataset) for feature selection. The entire training dataset was used for PDF/PMF estimation. The overall setup is shown in Figure 4(a).

In order to build the classifier, we first evaluated the discriminative power of each pathway on the marker-evaluation dataset. The pathways were sorted according to their absolute *t*-test statistics score in a descending order and the top 50 pathways were selected as potential features. Initially, we started with an LDA-based classifier with a single feature (i.e., the pathway marker that is on the top of the list) and continued to expand the feature set by considering additional pathway markers in the list. The classifier was trained using the marker-evaluation dataset and its performance was assessed on the feature-selection dataset by measuring the AUC. Pathway markers were added to the feature set only when they increased the AUC. Finally, the performance of the classifier with the optimal feature set was evaluated by computing the AUC on the testing dataset. The above process was repeated for 100 random partitions to ensure reliable results, and we report the average AUC as the measure of overall classification performance.

**Figure 5:**
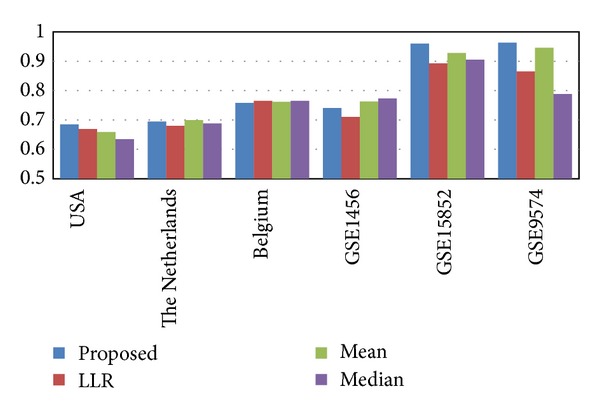
Classification performance for within-dataset experiments. The bars show the classification performance (average AUC) of different pathway activity inference methods evaluated on various breast cancer datasets.

Figure 5 shows how the respective classifiers that use different pathway activity inference methods perform on different datasets. As we can see in Figure 5, among the four inference methods, the proposed rank-based scheme typically yields the best average performance across these datasets. We also performed similar experiments based on the USA and the Belgium datasets after normalizing the raw data using different normalization methods. These results are summarized in Figure S5. We can see from Figure S5 that the proposed method yields the best performance on the USA dataset for all three normalization methods. On the Belgium dataset, the proposed method yields good consistent performance that is not very sensitive to the normalization method.

### 3.3. Reproducibility of the Pathway Markers Identified by the Proposed Method

To assess the reproducibility of the pathway markers, we performed the following cross-dataset experiments based on a similar setup that has been utilized in previous studies [8–11]. In this experiment, we used one of the breast cancer datasets for selecting the best pathway markers (i.e., only for feature selection) and a different dataset for building the classifier (using the selected pathways) and evaluating the performance of the resulting classifier. More specifically, we proceeded as follows. The first dataset was first divided into threefolds, where twofolds were used for marker evaluation and the remaining fold was used for feature selection. The second dataset was randomly divided into fivefolds, where fourfolds were used to train the LDA classifier, using the features selected from the first dataset, and the remaining fold was used to evaluate the classification performance. The overall setup is shown in Figure 4(b). To obtain reliable results, we repeated this experiment for 100 random partitions (of the second dataset) and report the average AUC as the performance metric. For these experiments, we used the three largest breast cancer datasets (USA, The Netherlands, and Belgium) among the six.

**Figure 6:**
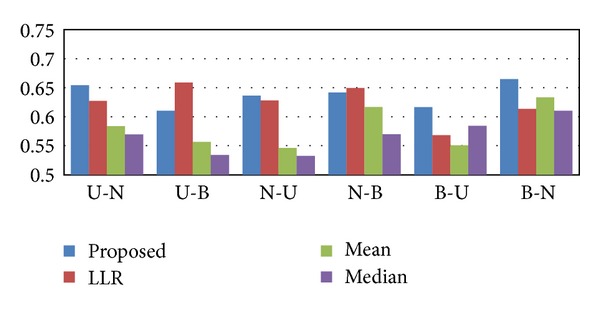
Classification performance for cross-dataset experiments. The bars show the cross-dataset classification performance (average AUC) of different pathway activity inference methods. The first dataset was used for selecting the pathway markers and the second dataset was used for training and evaluation of the classifier. The three largest breast cancer datasets were used: USA (U), The Netherlands (N), and Belgium (B).

**Figure 7:**
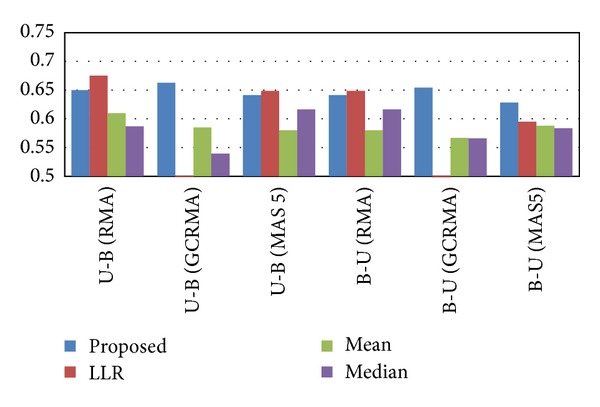
Classification performance for cross-dataset experiments. We repeated the cross-dataset experiments based on the USA and the Belgium datasets after normalizing the raw data using different normalization methods.

The results of the cross-dataset classification experiments are shown in Figure 6. As we can see from this figure, the proposed rank-based inference scheme typically outperforms other methods in terms of reproducibility. Furthermore, we can also observe that the proposed method yields consistent classification performance across experiments, while the performance of other inference methods is much more sensitive on the choice of the dataset. Next, we repeated the cross-dataset classification experiments based on the USA and the Belgium datasets after normalizing the raw data using RMA, GCRMA, and MAS5. As shown in Figure 7, the proposed method yields consistently good performance, regardless of the normalization method that was used.

Finally, we performed additional cross-dataset experiments after normalizing the USA and the Belgium datasets using different normalization methods. These results are summarized in Figures S6 and S7. We can see that the proposed pathway activity inference scheme is relatively robust to “normalization mismatch.” Moreover, these results also show that the proposed scheme overcomes the problem of the previous LLR-based scheme [10] when used with GCRMA (see Figures 7, S6, and S7).

## 4. Conclusions

In this work, we proposed an improved pathway activity inference scheme, which can be used for finding more robust and reproducible pathway markers for predicting breast cancer metastasis. The proposed method integrates two effective strategies that have been recently proposed in the field: namely, the probabilistic pathway activity inference method [10] and the ranking-based relative gene expression analysis approach [3]. Experimental results based on several breast cancer gene expression datasets show that our proposed inference method identifies better pathway markers that have higher discriminative power, are more reproducible, and can lead to better classifiers that yield more consistent performance across independent datasets.

## Supplementary Material

The Supplementary Material contains figures related to the experimental results based on the USA and Belgium datasets normalized using different normalization methods. Figure S1 contains the discriminative power of pathway markers on the same dataset. Figures S2–S4 contains the discriminative power of pathway markers across different datasets. The classification results of within-dataset experiments are in Figure S5, and the classification results of cross-dataset experiments are in Figures S6 and S7.
