# Supplementary material for: Identification of Robust Pathway Markers for Cancer through Rank-Based Pathway Activity Inference
Source: Adv Bioinformatics. 2013 Feb 27;2013:618461. doi: 10.1155/2013/618461 (PMC3600350; doi:10.1155/2013/618461)
Supplement: Supplementary file 1 — The Supplementary Material contains figures related to the experimental results based on the USA and Belgium datasets normalized using different normalization methods. Figure S1 contains the discriminative power of pathway markers on the same dataset. Figures S2–S4 contains the discriminative power of pathway markers across different datasets. The classification results of within-dataset experiments are in Figure S5, and the classification results of cross-dataset experiments are in Figures S6 and S7. [file 618461.f1.pdf]

Supplementary Material for "Identification of Robust Pathway Markers for Cancer Through Rank-Based Pathway Activity Inference"

Navadon Khunlertgit[1], Byung-Jun Yoon[1,*]

1 Department of Electrical and Computer Engineering, Texas A&M University,

College Station, TX 77843-3128, USA.

∗ **E-mail: bjyoon@ece.tamu.edu**

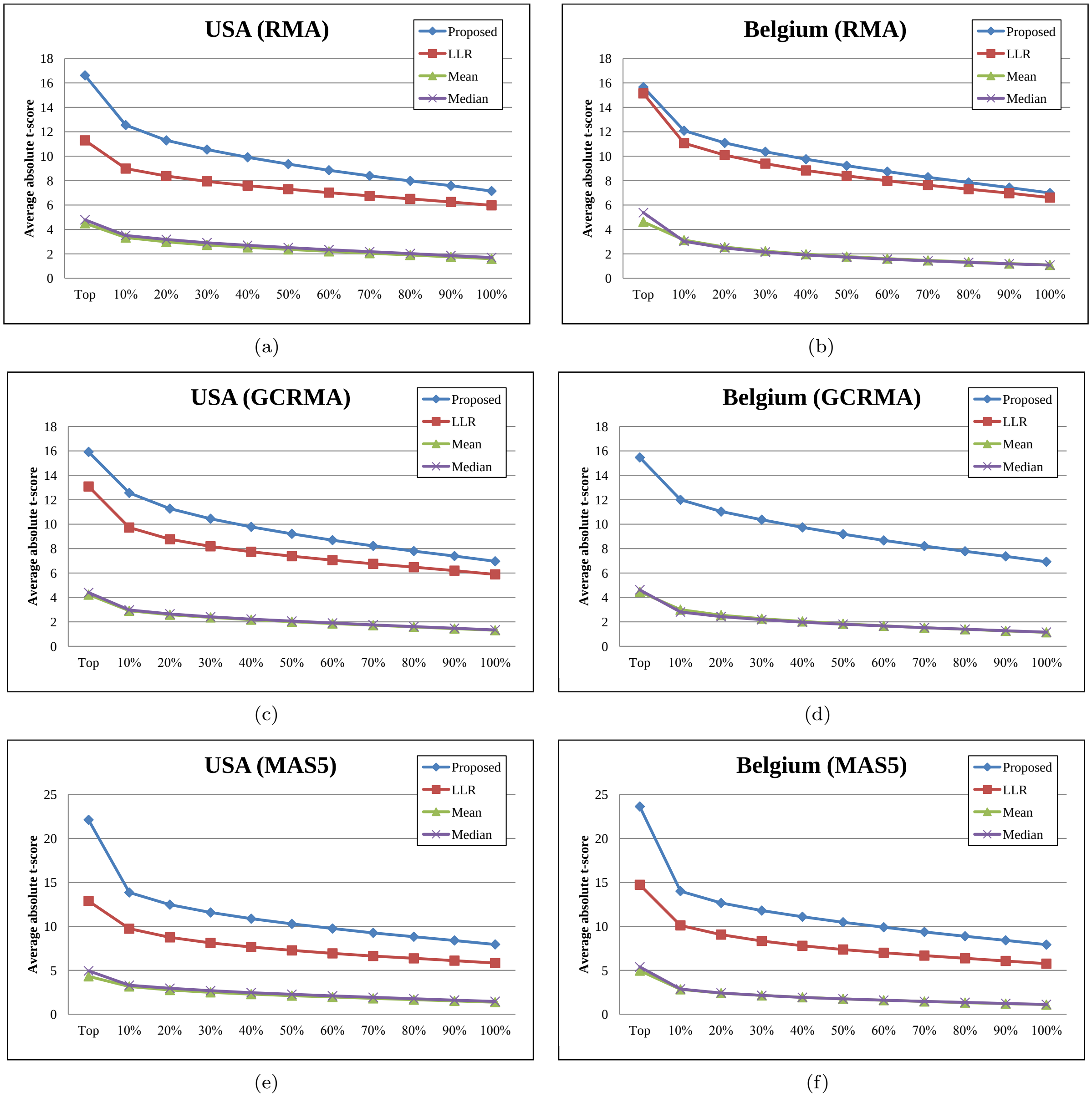

Figure S1. Discriminative power of pathway markers. We computed the mean absolute t-score of the top P% markers for the USA and the Belgium datasets after normalizing the raw data using three different normalization methods: RMA, GCRMA, and MAS5.

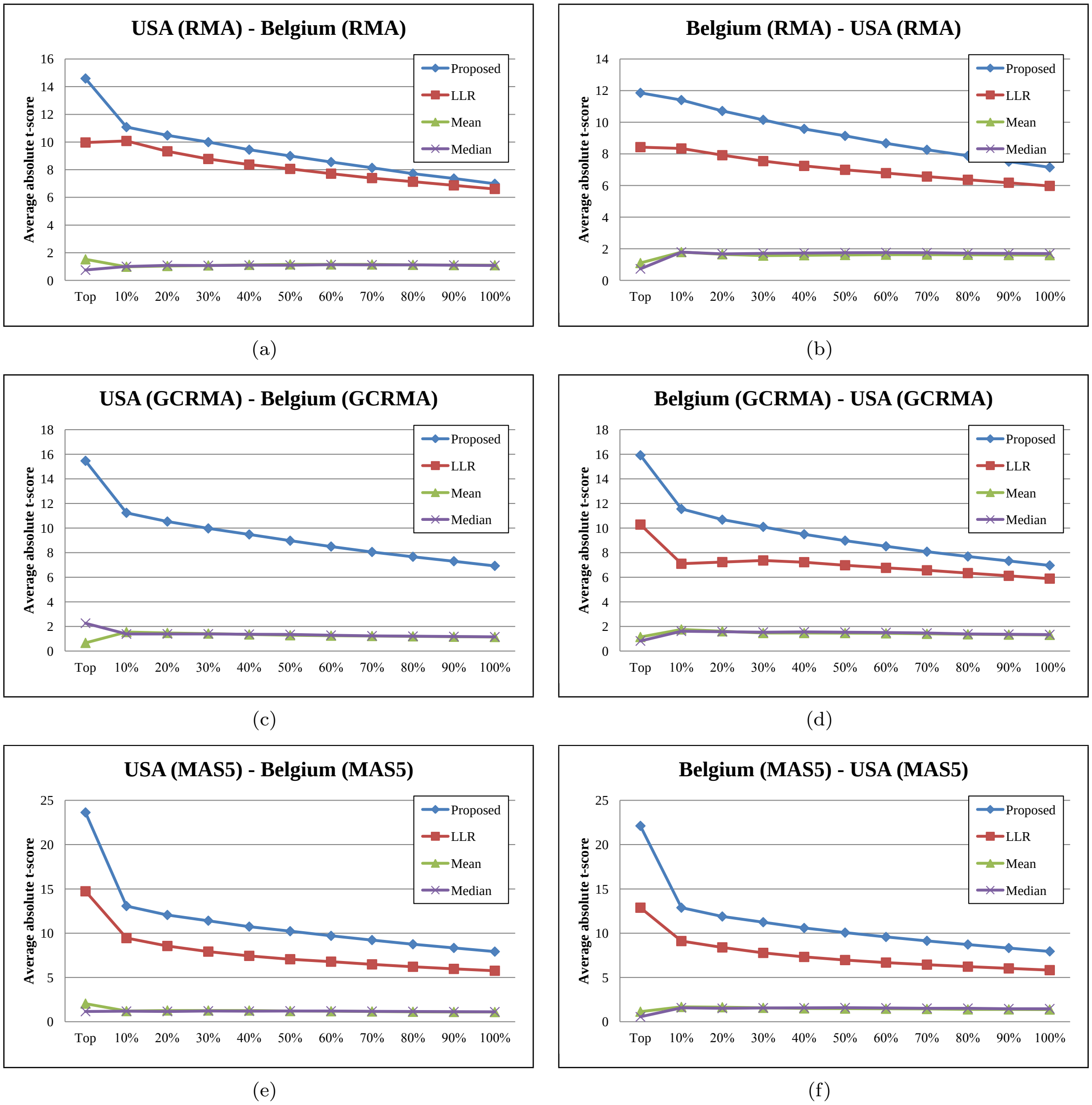

Figure S2. Discriminative power of pathway markers across different datasets. The pathway markers have been ranked and sorted using the first dataset and their discriminative power has been re-evaluated using the second dataset. In all experiments, the datasets have been first normalized using the same normalization method.

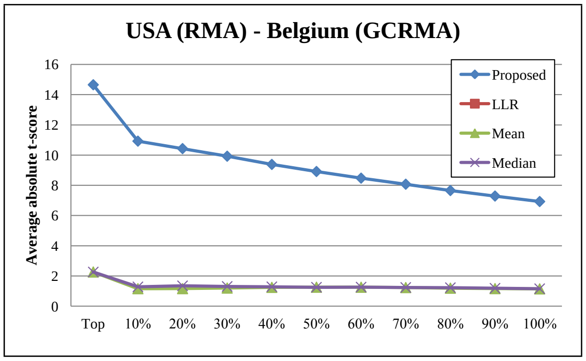

(a)

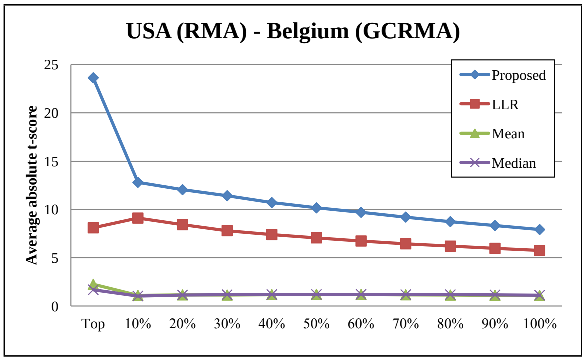

(b)

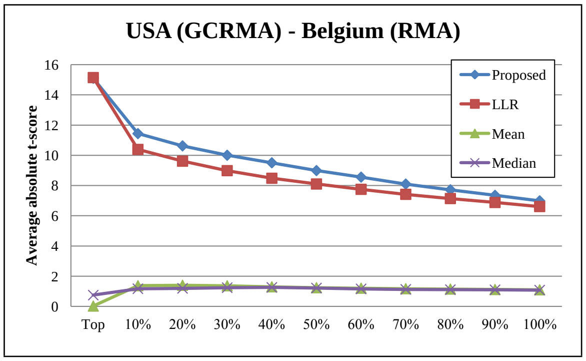

(c)

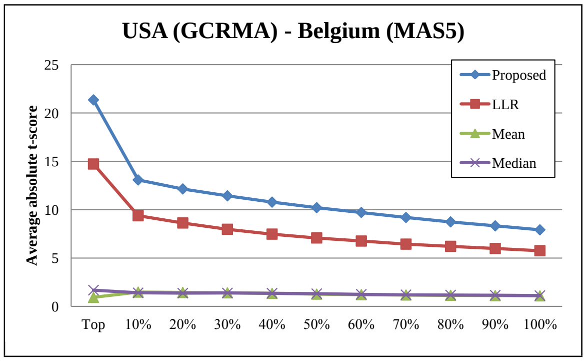

(d)

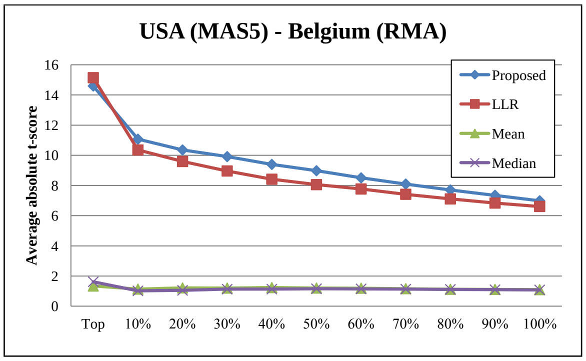

(e)

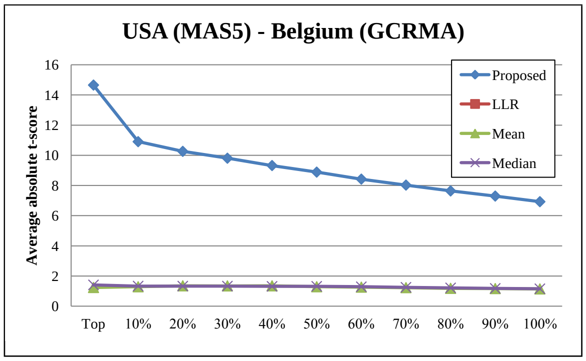

(f)

Figure S3. Discriminative power of pathway markers across different datasets. The pathway markers have been ranked and sorted using the USA dataset and their discriminative power has been re-evaluated using the Belgium dataset. In these experiments, the two datasets have been normalized using different normalization methods.

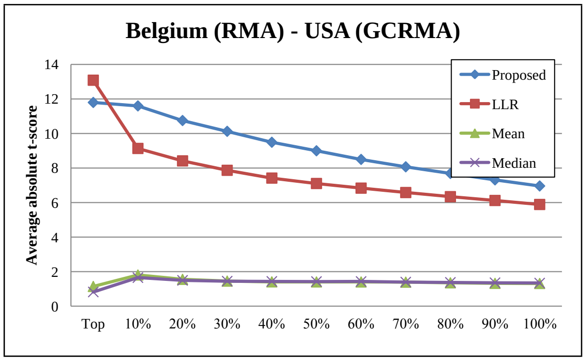

(a)

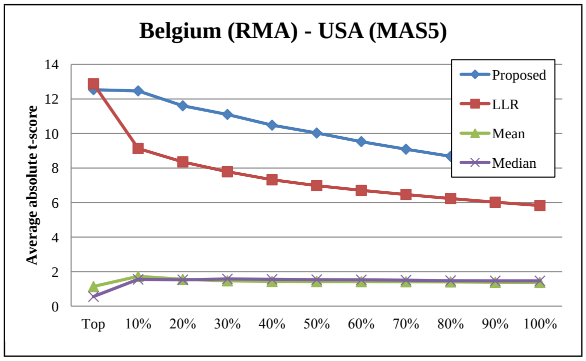

(b)

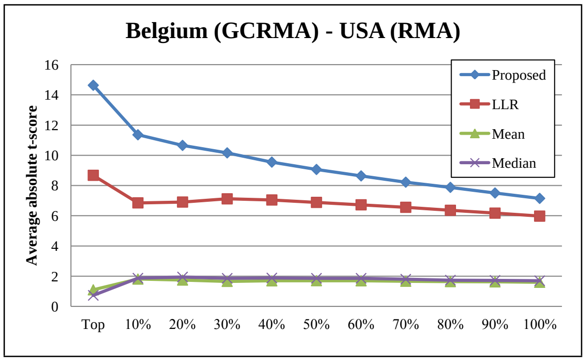

(c)

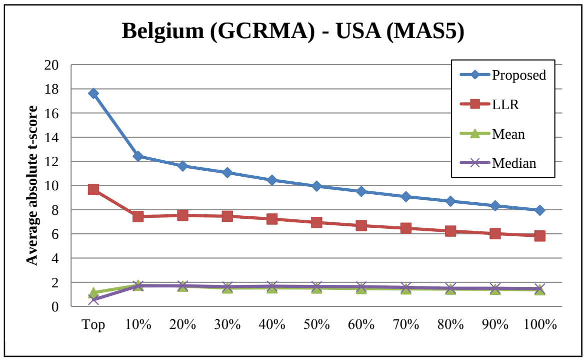

(d)

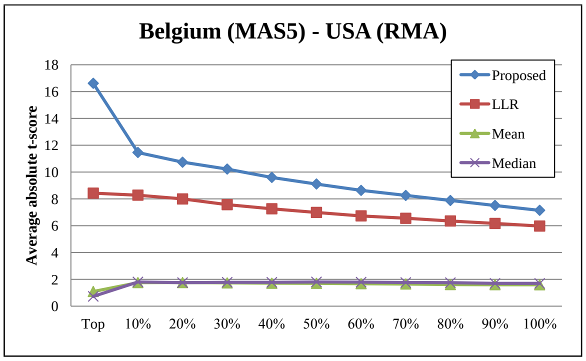

(e)

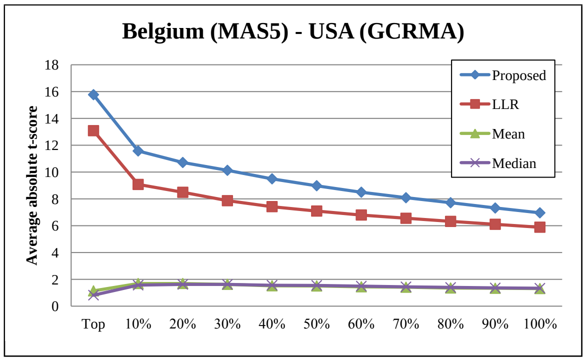

(f)

Figure S4. Discriminative power of pathway markers across different datasets. The pathway markers have been ranked and sorted using the Belgium dataset and their discriminative power has been re-evaluated using the USA dataset. In these experiments, the two datasets have been normalized using different normalization methods.

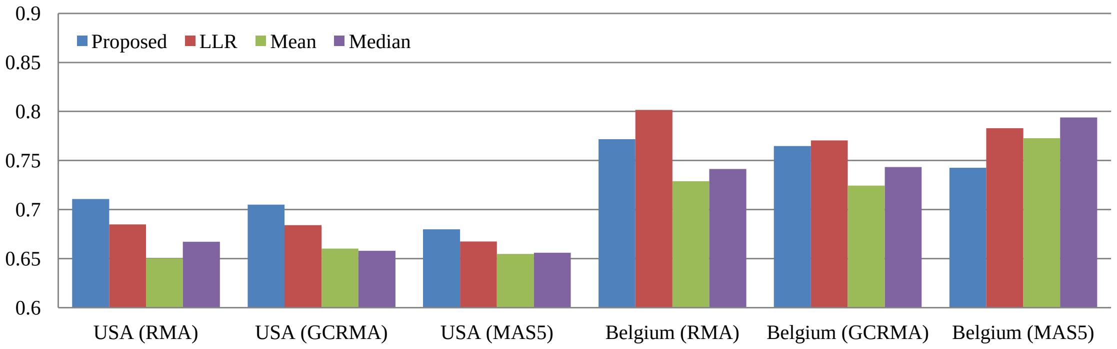

Figure S5. Classification performance for within-dataset experiments. We repeated the within-dataset classification experiments based on the USA and the Belgium datasets after normalizing the raw data using three different normalization methods: RMA, GCRMA, and MAS5.

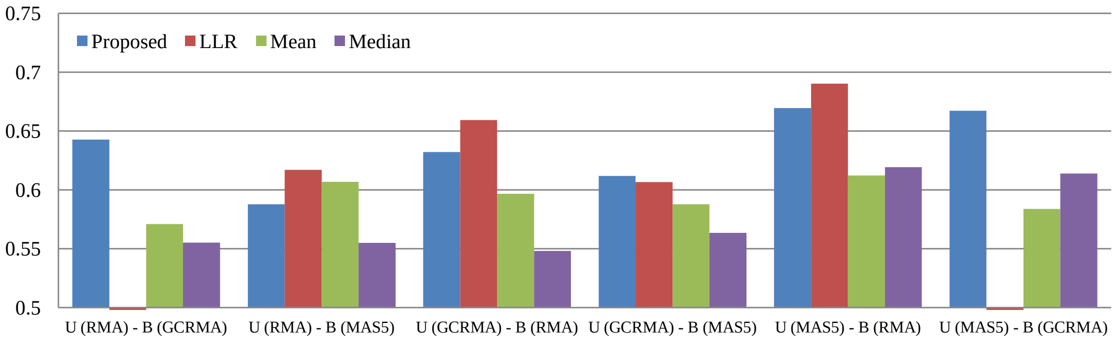

Figure S6. Classification performance for cross-dataset experiments. The USA dataset was used to select the pathway markers and the Belgium dataset was for training and evaluating the classifier. In these experiments the two datasets have been normalized using different normalization methods.

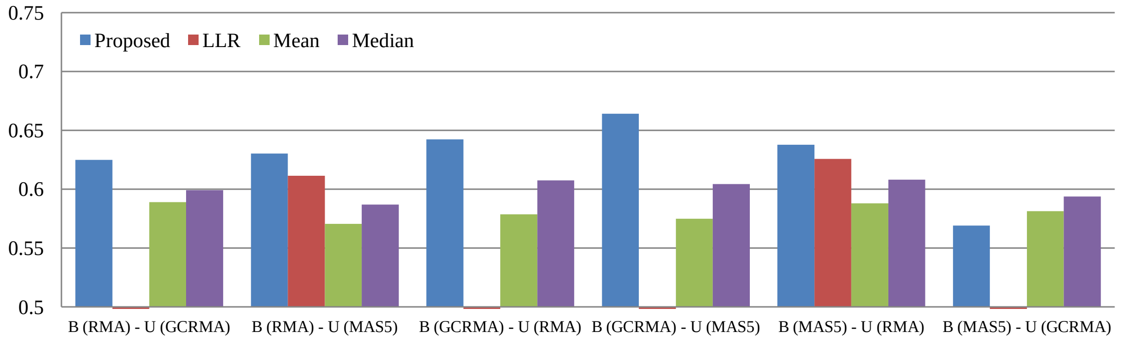

Figure S7. Classification performance for cross-dataset experiments. The Belgium dataset was used to select the pathway markers and the USA dataset was for training and evaluating the classifier. In these experiments the two datasets have been normalized using different normalization methods.
